# Drowning deaths in Sweden with emphasis on the presence of alcohol and drugs – a retrospective study, 1992–2009

**DOI:** 10.1186/1471-2458-13-216

**Published:** 2013-03-11

**Authors:** Kristin Ahlm, Britt-Inger Saveman, Ulf Björnstig

**Affiliations:** 1 Section of Forensic Medicine, Department of Community Medicine and Rehabilitation, Umeå University, POB 7616, Umeå, SE 907 12, Sweden; 2 Department of Nursing, Umeå University, Umeå, SE 901 87, Sweden; 3 Division of Surgery/KBC, Department of Surgical and Perioperative Sciences, Umeå University, Umeå, SE 901 87, Sweden

**Keywords:** Alcohol, Drowning, Illicit drugs, Pharmaceutical drugs, Suicide

## Abstract

**Background:**

Drowning deaths constitute a significant proportion of unnatural deaths globally. In Sweden and other high-income countries, drowning deaths have decreased. This study investigates the epidemiology and current trends of unintentional, intentional, and undetermined drowning deaths with emphasis on the presence of alcohol and other drugs.

**Methods:**

During an 18-years period, 5,125 drowning deaths were autopsied in Sweden. Data on cases including toxicological analysis on alcohol, pharmaceutical drugs, and illicit drugs were obtained from the National Board of Forensic Medicine.

**Results:**

During the study period, the annual incidence of drowning deaths in Sweden was 3.1/100,000 inhabitants and decreased on average by about 2% each year (p<0.001). The highest incidence was found among males and in middle/older age groups. The incidence increased 3% for each year of age. Children/adolescents (≤18 years) constituted 5% of all drowning deaths. Of all drowned females in the study, 55% (847/1,547) committed suicide, which was a significantly higher proportion compared with males (21%, 763/3,578) (p<0.001). In total, 38% (1,656/4,377) of tested drowned persons had alcohol in their blood and the mean concentration was 1.8 g/l. In the unintentional drowning group, intentional drowning group, and the undetermined group, the proportion of alcohol positive was 44%, 24%, and 45%, respectively. One or several psychoactive drugs were present in the blood in 40% (1,688 /4,181) of all tested persons and in 69% (965/1,394) of tested persons who died from suicidal drowning. The most common drug was benzodiazepines (21%, 891/4,181). Illicit drugs were detected in 10% (82/854) of tested persons.

**Conclusion:**

Presence of alcohol and drugs were frequent and may have contributed to the drowning deaths. The incidence of drowning deaths significantly decreased during the study period. Males and the middle/older age groups had a higher incidence compared to females and children. Suicidal drowning was common especially among women. Alcohol and drugs are significant contributors in drowning deaths in Sweden and should be considered as part of a comprehensive prevention program.

## Background

Drowning is the third most frequent cause of unnatural deaths in the world. In 2004, 388,000 persons died of drowning [1]. Drowning mortality rates are higher in low-income countries [2,3] and among vulnerable/underprivileged groups [4]. Globally, drowning deaths is common among children, a group at elevated risk [2]. The incidence of drowning deaths for males is twice as high as for females [1].

A population-based study from Finland (1970–2000) revealed that the WHO statistics underestimated the real number of drowning deaths by 40-50% [5]. The discrepancy could partly be explained by the fact that WHO did not include drowning deaths associated with traffic accidents, boating accidents, or natural disasters (e.g., floods) [5].

Drowning deaths still constitutes a significant proportion of unnatural deaths in Sweden as well as in other high-income countries. Although there has been a decrease in the number of drowning deaths (especially among children), it is unclear whether the incidence is still high in some groups. In Sweden (9.5 million inhabitants), drowning deaths account for 6% of all unnatural deaths [6].

Drowning deaths can occur during various water activities such as swimming, bathing, boating, and in incidents with motor vehicles [5,7,8]. Another common circumstance is suicide, often related to a previously known psychiatric illness [9]. Disease, such as epilepsy, may also play a role in drowning [10,11]. Alcohol and drugs often contribute to drowning deaths [5,8,12,13]. Therefore, increased knowledge about the circumstances and the role of alcohol and drugs in drowning deaths is needed to develop effective preventive measures in different subgroups. In addition, it is important to analyse trends to understand whether this problem is increasing or decreasing. Only a few studies include the circumstances and intention of drowning deaths especially in relation to the influence of alcohol and other drugs [5,13]. This study analyses the epidemiology and current trends of unintentional, intentional, and undetermined drowning deaths in Sweden with emphasis on the presence of alcohol and other drugs.

## Methods

This study includes persons who died of drowning in Sweden from 1 January 1992 through 31 December 2009 (n = 5,125). Information on these cases with the ICD-9 code 994.1 [14] was obtained from the Forensic Medicine Database of the National Board of Forensic Medicine. As the law recommends autopsies be performed for cases of unnatural deaths, a complete autopsy is the routine in these cases.

This database includes information on age, sex, circumstances, cause of death, and manner of death (unintentional, intentional, or undetermined). Out of 5,125 cases, 169 individuals who drowned were not permanent residents in Sweden, so they were not included when calculating the incidence of drowning per 100,000 inhabitants in Sweden. In addition, in seven unidentified cases the age of the deceased was missing.

Data on alcohol and other toxicological substances were obtained from Forensic Toxicology, National Board of Forensic Medicine, in Linköping, Sweden. Analyses of alcohol, pharmaceutical drugs, and illicit drugs in femoral vein blood were performed using headspace gas chromatography [15,16].

The pharmaceuticals were grouped according to the WHO’s Anatomical Therapeutic Chemical (ATC) Classification System [17]: psychoactive drugs; benzodiazepines, opiates, neuroleptics, antiepileptic, and other drugs. Testing for illicit drugs, unlike testing for alcohol, is not a routine practice at autopsy in Sweden. In each case, the forensic pathologist weights all available information and decides whether such a test should be done. To avoid overestimating the results on alcohol and other drugs, 313 cases were excluded because the body was decomposed. Blood alcohol concentration (BAC) below <0.2 g/l was considered to be negative in this study since the alcohol level can increase due to decomposition [18]. In the remaining 4,812 cases, presence of alcohol and drugs was analysed.

Permission to use the autopsy and toxicological data for this study was obtained from the National Board of Forensic Medicine.

### Statistical methods

Annual incidence of drowning deaths for different age groups, males, and females were calculated and presented as cases per 100,000 of mean population. A Poisson regression model was used to analyse time trend, age effects, and differences between males and females. Results from the Poisson regression model were given as incidence rate ratios (IRR) and 95% confidence intervals (CI). Comparison of differences between the groups unintentional and intentional drowning with respect to alcohol use and sex was done using the Chi-square test performed in Epi Info 3.4. SPSS 19 was used for other statistical calculations.

## Results

During the 18-year study period, 5,125 individuals drowned and were autopsied and the mean incidence was 3.1/100,000 inhabitants. This incidence decreased by about 2% each year (CI 0.98-0.99, p<0.001): males from 5.6 to 3.1 and females from 2.8 to 1.3/100,000 inhabitants. The decrease varied from 1-3% in the different age groups. Forensic Medicine in Sweden is divided in six catchment areas, each served by a regional Department of Forensic Medicine. The average annual incidence of drowning deaths varied between 2.9-3.9/100,000 inhabitants with the highest rate in the northern district (Umeå) (Table 1). Drowning deaths defined as unintentional were more common in the northern district (incidence 2.7/100,000) compared with the other districts (1.3-1.9/100,000), and there was a higher proportion of drowning associated with thin ice (16% compared to 1-6%), boat incidents (22% compared to 9-10%), and presence of alcohol (46% compared to 30-41%).

**Table 1:** The incidence of drowning deaths in Sweden in relation to the catchment areas of Departments of Forensic Medicine (1992–2009)

**Catchment area**	**Number of drowning deaths**	**Population**	**Incidence per 100,000 inhabitants**
Stockholm	1112	2144 840	2.9
Uppsala	663	1119 604	3.3
Linköping	915	1534 983	3.3
Lund	861	1594 261	3.0
Göteborg	933	1664 357	3.1
Umeå	641	895 264	3.9

**Figure 1:**
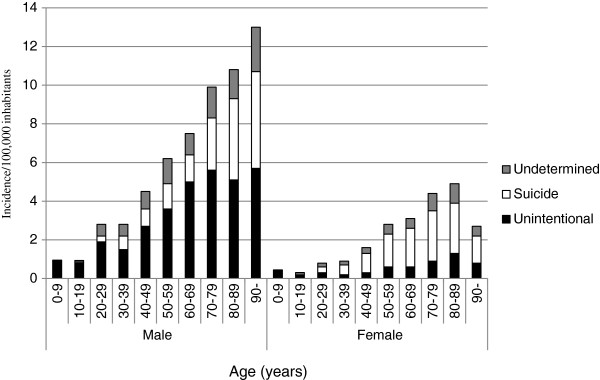
**Incidence of drowning deaths per 100,000 inhabitants in relation to age, sex, and manner of death in Sweden from 1992 through 2009.** (Homicide drowning (n=21) is not included).

**Figure 2:**
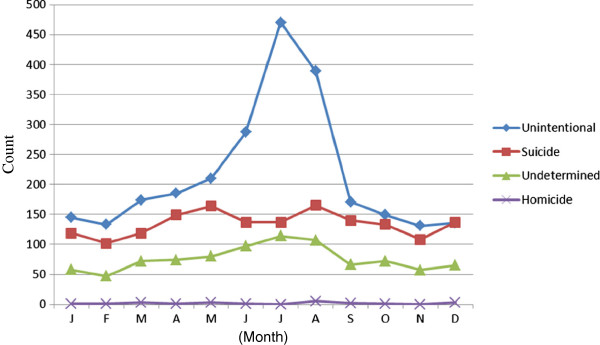
Number of drowning deaths in relation to months and manner of death in Sweden from 1992 through 2009 (n = 5,125).

There was a male predominance in all age groups (IRR 2.5 compared to females, CI 2.4-2.7, p<0.001). Of the 5,125 drowning deaths, 3,578 (70%) were male with a mean age of 53 years (range 0–101 years, SD 20). The mean age of the females was 58 years (range 0–96 years, SD 20). Of all drowning deaths, 5% (n=248) were ≤ 18 years old. For both sexes, the incidences increased with age (Figure 1). The incidence of drowning deaths increased on average by 3% for each year of age (CI 1.03-1.03, p< 0.001). Seasonal variation was noted in the unintentional drowning category with a peak during the summer months (June through August) and many of these drowning deaths were related to boating, bathing, or other outdoor water activities (Figure 2).

### Unintentional drowning

Unintentional drowning caused 2,585 (50%) deaths – 2,180 (84%) were males and 405 (16%) females. The mean age for males was 54 years (range 1–101, SD 22), and the mean age for females was 56 years (range 0–93, SD 26). The highest numbers of drowning deaths were in age groups 50–59 and 60–69 years (17% and 18%, respectively). Most unintentional drowning occurred in lakes, sea, streams or rivers, or bathtubs (Table 2). Thin ice was associated with 262 (13%) drowning incidents that occurred in lakes, seas, streams, and rivers.

**Table 2:** Drowning deaths in Sweden in relation to manner of death, location, sex, and presence of alcohol in blood (1992–2009)

**Localization of drowning**	**Male N (%)**		**Female N (%)**		**Total N (%)**		**Alcohol N positive/ N tested (%)**	
**Unintentional**	**2180**	**(100%)**	**405**	**(100%)**	**2585**	**(100%)**	**997/2255**	**(44%)**
Bathtub	177	(8)	139	(34)	316	(12)	90/257	(35)
Pool, pond	93	(4)	18	(4)	111	(4)	28/84	(33)
Sea	566	(26)	74	(18)	640	(25)	261/576	(45)
Lake	744	(34)	81	(20)	825	(32)	316/743	(43)
Stream, river	456	(21)	65	(16)	521	(20)	253/449	(56)
Other places	60	(3)	8	(2)	68	(3)	20/63	(32)
Data not available	84	(4)	20	(5)	104	(4)	29/83	(35)
**Intentional**								
***Suicide***	**763**	**(100%)**	**847**	**(100%)**	**1610**	**(100%)**	**333/1400**	**(24%)**
Bathtub	153	(20)	318	(38)	471	(29)	131/429	(31)
Pool, pond	17	(2)	15	(2)	32	(2)	4/28	(14)
Sea	163	(21)	129	(15)	292	(18)	64/243	(26)
Lake	167	(22)	159	(19)	326	(20)	61/287	(21)
Stream, river	213	(28)	192	(23)	405	(25)	64/334	(19)
Other places	43	(6)	26	(3)	69	(4)	5/64	(8)
Data not available	7	(0.9)	8	(0.9)	15	(0.9)	4/15	(27)
***Homicide***	**12**	**(100%)**	**9**	**(100%)**	**21**	**(100%)**	**7/19**	**(37%)**
Bathtub	3	(25)	3	(33)	6	(29)	2/5	(40)
Pool, pond	1	(8)	0	(0)	1	(5)	1/1	(100)
Sea	2	(17)	3	(33)	5	(24)	0/5	(0)
Lake	2	(17)	1	(11)	3	(14)	1/3	(33)
Stream, river	1	(8)	1	(11)	2	(10)	1/1	(100)
Data not available	3	(25)	1	(11)	4	(19)	2/4	(50)
**Undetermined**	**623**	**(100%)**	**286**	**(100%)**	**909**	**(100%)**	**319/703**	**(45%)**
Bathtub	95	(15)	116	(41)	211	(23)	71/168	(42)
Pool, pond	16	(3)	3	(1)	19	(2)	8/17	(47)
Sea	147	(24)	47	(16)	194	(21)	78/142	(55)
Lake	151	(24)	40	(14)	191	(21)	64/148	(43)
Stream	186	(30)	73	(26)	259	(28)	88/198	(44)
Other places	12	(2)	2	(0.7)	14	(2)	4/11	(36)
Data not available	16	(3)	5	(2)	21	(2)	6/19	(32)
Total	3578		1547		5125		1656/4377	(38)

Unintentional drowning deaths were associated with bathing and other unspecified water activities (1807, 70%), boating incidents (521, 20%), cars driving into water (82, 3%), snowmobiles (78, 3%), diving (51, 2%), and airplanes, helicopters, and other vehicles (46, 2%).

### Intentional drowning

#### Suicide

During the study period, there were 1,610/5,125 (31%) suicides by drowning (47% males and 53% females); the mean age for males was 59 years (range 16–97 year, SD 18) and 61 years for females (range 14–96 year, SD 18). Of all female drowning deaths, 55% (847/1,547) committed suicide, which was significantly higher compared to males (21%, 763/3,578, p<0.001). For both sexes, the highest incidence of suicidal drowning was found in age groups 50–59 and 60–69 years (in females 1.7 and 2.0/100,000, respectively, and in males 1.3 and 1.4/100,000, respectively). During the study period, the incidence of all suicidal drowning deaths decreased for females from 1.6 to 0.7 and for males from 1.2 to 0.8 per 100,000 inhabitants. Most suicidal drowning deaths were found in water outdoors (Table 2), and for the majority the preceding activity was unknown. In 61 (4%) of all suicidal drowning deaths, the person jumped from a bridge or hill into water or 29 (2%) drove a car into water. Compared with other locations, suicidal drowning deaths occurred more frequently in a bathtub for females than for males (38% versus 20%) (p< 0.01) (Table 2).

#### Homicide

There were 21 identified homicides – twelve (57%) males and nine (43%) females. The median age for males was 27 years (range 1–81, SD 22) and for females, 16 years (0–87, SD 30). Seven children/adolescents (0–16 years) were murdered. For all homicides, the bathtub (29%) and the sea (24%) were the most prevalent sites (Table 2).

### Undetermined drowning

In 909/5,125 cases (18%), intent was unable to establish. This group mainly consisted of males (623, 68%). The mean age for males was 53 years (range 0–93, SD 19) and the mean age for females was 59 years (range 0–93, SD 20). Incidence increased with age for both males and females (Figure 1). Stream, river, bathtubs, sea, and lakes were the most frequent drowning locations. Significantly more females drowned in a bathtub compared to males also in this group (p<0.01) (Table 2).

### Alcohol

In total, 4,377 out of 4,812 (91%) individuals were tested for alcohol and 1,656 (38%) had BAC ≥ 0.2 g/l in blood with a mean BAC of 1.8 g/l (range 0.2-5.2). The presence of alcohol was more frequent in unintentional drowning deaths compared to suicidal drowning deaths (p< 0.001) (Table 2) and in males compared to females (p<0.01) (Table 3).

**Table 3:** Presence of alcohol, pharmaceutical drugs, and illicit drugs in relation to sex among drowning deaths in Sweden (1992–2009)

	**Male N (%)**		**Female N (%)**		**Total N (%)**	
**Blood alcohol**						
Negative*	1756	(58)	965	(72)	2721/4377	(62)
Positive	1285	(42)	371	(28)	1656/4377	(38)
**BAC (g/l)**						
0.2-0.4	111	(9)	46	(12)	157	(9)
0.5-0.9	116	(9)	58	(16)	174	(11)
1.0–1.4	137	(11)	53	(14)	190	(11)
1.5–1.9	244	(19)	71	(19)	315	(19)
2.0–2.4	273	(21)	55	(15)	328	(20)
2.5–2.9	254	(20)	54	(14)	308	(19)
3.0–3.4	103	(8)	19	(5)	122	(7)
≥3.5	47	(4)	15	(4)	62	(4)
*Not tested*	*287/3041*	*(9)*	*151/1336*	*(11)*	*435/4812*	*(9)*
**Pharmaceutical**						
Negative	1949/2863	(68)	477/1318	(36)	2426/4181	(58)
Positive	914/2863	(32)	841/1318	(64)	1755/4181	(42)
Benzodiazepines	384/2863	(13)	507/1318	(38)	891/4181	(21)
Opiates	241/2863	(8)	229/1318	(17)	470/4181	(11)
Antidepressant	410/2863	(14)	452/1318	(34)	612/4181	(15)
Neuroleptica	173/2863	(6)	165/1318	(13)	338/4181	(8)
Antiepileptic	53/2863	(2)	28/1318	(2)	81/4181	(2)
*Not tested*	*462/3325*	*(14)*	*169/1487*	*(11)*	*631/4812*	*(13)*
**Illicit drugs**						
Negative	577/650	(89)	195/204	(96)	772/854	(90)
Positive	73/650	(11)	9/204	(4)	82/854	(10)
*Not tested*	*2675/3325*	*(80)*	*1283/1487*	*(86)*	*3958/4812*	*(82)*

* BAC below < 0.2 ‰ was considered to be negative since alcohol level can raise due to decomposition process.

#### Unintentional drowning

Alcohol was found in 997/2,255 (44%) of the tested individuals in the unintentional drowning group (Table 4) and 777 of them (78%) had a BAC of 1.5 g/l and higher (Table 3). A higher proportion of males than females had alcohol in their blood (p<0.01). For both males and females, the age groups between 30 and 69 years had the highest number of alcohol positive cases (Table 4). The proportion of positive blood alcohol was highest for individuals who drowned in streams and rivers (p<0.001) (Table 2). Alcohol was detected in 256 (54%) of 471 tested individuals who fell from a boat. Of the diving incidents, all 44 individuals tested negative. Fifty (68%) of 73 snowmobile riders who drowned after breaking through thin ice were alcohol positive. In drowning incidents with other motor vehicles, 35 (30%) of 115 occupants had alcohol in their blood.

**Table 4:** Presence of alcohol in individuals in relation to manner of death, sex, and age among drowning deaths in Sweden (1992–2009)

	**Unintentional**		**Intentional**				**Undetermined**	
			**Suicide**		**Homicide**			
**Sex**	**N**	**(%)**	**N**	**(%)**	**N**	**(%)**	**N**	**(%)**
Males	881/1916	(46)	158/647	(24)	4/10	(40)	248/468	(53)
Females	116/339	(34)	175/753	(23)	3/9	(33)	77/235	(33)
**Age groups (years)**	**N**	**(%)**	**N**	**(%)**	**N**	**(%)**	**N**	**(%)**
0-9	2/52	(4)	0	(0)	0/2	(0)	0	(0)
10-19	27/95	(28)	7/13	(54)	3/5	(60)	7/8	(88)
20-29	99/221	(45)	33/53	(62)	2/2	(100)	36/56	(64)
30-39	102/202	(50)	44/112	(39)	0/2	(0)	38/67	(58)
40-49	187/317	(59)	53/174	(30)	1/3	(33)	56/96	(58)
50-59	269/411	(65)	88/274	(32)	1/2	(50)	86/154	(56)
60-69	213/414	(51)	44/251	(18)	0	(0)	55/109	(50)
70-79	78/347	(22)	43/297	(14)	0/1	(0)	33/126	(26)
80-89	17/171	(10)	18/200	(9)	0/2	(0)	8/70	(11)
>90	2/24	(8)	3/26	(12)	0	(0)	0	(0)
	**N**	**(%)**	**N**	**(%)**	**N**	**(%)**	**N**	**(%)**
**Total**	996/2254*	**(44)**	**333/1400**	**(24)**	**7/19**	**(37)**	**319/703**	**(45)**

*One individual was not identified and the age missing.

#### Intentional drowning

Of those who committed suicide, 333 (24%) of 1,400 tested individuals had alcohol in their blood (Table 4). There was no significant difference in proportion of alcohol between males and females (p= 0.605). Younger age groups had higher proportion of alcohol (Table 4). The highest proportion of alcohol was found in individuals who drowned in bathtubs (p<0.01, Table 2). Alcohol was detected in 16 (37%) of the 43 tested who jumped from a bridge, 13 (52%) of the 25 who committed suicide by driving a car into water, and four (50%) of eight who committed suicide using boats. Of the 21 homicides, alcohol was found in 37% of the cases (Table 4).

#### Undetermined drowning

In the undetermined drowning group, 319 (45%) of 703 tested individuals had alcohol in their blood (Table 2). Significantly more males than females had alcohol in their blood (p< 0.001). The age groups 10–19 and 20–29 years had the highest proportion of alcohol in their blood (Table 4). More than half of those who drowned at sea tested positive for alcohol (Table 2).

### Pharmaceutical and illicit drugs

In all, 4,181 out of 4,812 (87%) individuals were tested for pharmaceutical substances in their blood (Table 3). In 1688 (40%) individuals, one or several psychoactive substances were detected. Multiple psychoactive drugs were found in 1,070 (26%) individuals. The most common psychoactive drugs were benzodiazepines and antidepressants (Table 3). The individuals who committed suicide had the highest proportion of pharmaceuticals compared with the undetermined and the unintentional groups (p< 0.001) (Table 5). The individuals between 50–79 years of age had the highest proportion of psychoactive substances (57%) in their blood (p<0.001). In 82 (10%) of 854 tested individuals, illicit drugs were detected in the blood (Table 3). A higher proportion (15%) of illicit drugs were found in the undetermined group compared to unintentional drowning deaths (9%) (p<0.001) (Table 5). The most common illicit drugs were amphetamine 42/82 (51%) and tetrahydrocannabinol 34/82 (41%).

**Table 5:** Presence of pharmaceutical and illicit drugs in relation to manner of death among drowning deaths in Sweden (1992–2009)

	**Unintentional**		**Intentional**				**Undetermined**	
			**Suicide**		**Homicide**			
**Pharmaceutical**	**N**	**(%)**	**N**	**(%)**	**N**	**(%)**	**N**	**(%)**
Negative	1613/2075	(78)	407/1394	(29)	15/17	(88)	391/695	(56)
Positive	462/2075	(22)	987/1394	(71)	2/17	(12)	304/695	(44)
*Not tested*	*403/2478*	*(16)*	*118/1512*	*(8)*	*4/21*	*(19)*	*106/801*	*(13)*
**Illicit drugs**	**N**	**(%)**	**N**	**(%)**	**N**	**(%)**	**N**	**(%)**
Negative	416/459	(91)	185/193	(96)	11/13	(85)	160/189	(85)
Positive	43/459	(9)	8/193	(4)	2/13	(15)	29/189	(15)
*Not tested*	*2019/2478*	*(81)*	*1319/1512*	*(87)*	*8/21*	*(38)*	*612/801*	*(76)*

#### Combination of alcohol and drugs

The combination of alcohol and psychoactive drugs was present in 518/4,083 (13%) and was more frequent in the suicide group (223/1,358, 16%) and the undetermined group (105/671 (16%) compared with the unintentional group (176/2,034, 9%) (p< 0.001). In 47 cases (6%) of those tested for illicit drugs, a combination of alcohol, pharmaceuticals, and illicit drugs was found.

## Discussion

The present study of unintentional, intentional, and undetermined drowning death showed a mean incidence of 3.1/100, 000 and an average overall decrease of 2% each year. In Sweden, the average annual number of drowning deaths during the study period was half of traffic deaths [19]. Notably, Australian researchers estimate that compared to traffic deaths the risk of drowning is 200 times higher when calculating the person-time exposures, indicating that drowning may merit interest from a prevention perspective [20].

The mean incidence of unintentional drowning deaths calculated from our Swedish death data was 1.6/100,000 inhabitants. In other Nordic countries, the corresponding figures range from 1.4/100,000 in Denmark to 6.1/100,000 inhabitants in Finland [5,21]. For 2005, the incidence was comparable with studies from Australia and the USA (1.3 and 1.2/100,000, respectively) [20,22].

Differences in incidence of drowning in various countries could be due to differences in demography, geography (e.g., presence of coast, lakes, and other water sources), the implementation of preventive measures (e.g., swimming skills, use of floating devices such as a life vest, using lifeguards to monitor public beaches, and fencing around pools), and risk taking behaviour [1,23]. Furthermore, different ways of collecting data and coding practices may also lead to differences in statistics [5,24]. From an international perspective, the incidence of drowning deaths among children was very low in the present study. However, older age groups still have a markedly high incidence of drowning, a finding also reported in previous studies from other countries [5,13]. This finding may be explained by more effective prevention for younger groups than for older age groups [13]. Especially in high-income countries several preventive measures have successfully been implemented for children and have led to a decrease in drowning deaths and hospitalization after drowning incidents [25].

There was a male predominance in unintentional drowning, a finding that previous studies also have noted [2,13,21,22,26,27]. The explanations for this might be that males participate in more water activities and are also likely to take more risks, especially after drinking alcohol [23]. In the present study, most unintentional drowning deaths occurred in lakes and at sea, but the most frequent location for females was a bathtub. The water location and activities of unintentional drowning cases vary according to access to sea and other water sources in different regions and countries [5,13,21]. It should be noted that there are approximately 520,000 lakes in Sweden [28]. The regional differences in Sweden especially regarding the incidence of unintentional drowning deaths could partly be explained by differences in access to water sources, leisure activities, and the length of ice coverage during winter, which in northern Sweden could last up to five months.

Drowning is a common method of suicide [9,29-32] and in the present study suicide constitutes 31% of all cases and 7% of all suicides in Sweden. Notably, in Sweden suicidal drowning is most frequent in females especially in the age groups 50 to 79 years. A similar result has been presented in a study from Croatia [33]. Suicide is a sign of psychiatric illness, which constitutes an important public health problem [34]. As other studies have noted [35,36], the suicide rate increases with age. The combination of depression and availability to bathtubs and other bodies of water may explain the high incidence of suicidal drowning. In the present study, psychoactive drugs were present in 71% of suicides, indicating that many of those individuals may have had contact with health care. The manner of suicide differs between males and females [35]; males tend to commit suicide in more violent ways and females tend to commit suicide by drowning or by overdosing on drugs [31,37]. Suicidal drowning and previous suicide attempts have a strong correlation, so it is particularly important to identify individuals suffering from depression and mental illnesses and initiate effective care and treatment to avoid suicides [38]. Health professionals dealing with suicidal cases need to be aware of the risk (especially for women) of drowning as a method to commit suicide often in combination with pharmaceutical drugs and alcohol.

Homicide due to drowning was uncommon in our study. However, in one-third of the homicides children (0-16 years of age) were the victims. The detailed circumstances could not be revealed in this study, but it has been reported that parents may kill their children by drowning them [39].

### Alcohol

A substantial proportion (44%) of unintentional drowning deaths tested positive for alcohol in the blood. This finding could be compared with 51% in Finland [5], 35%-55% in the USA [40], 50% in New Zealand [41], 62% in Ireland [32], and 22% in Australia [13]. The proportion of alcohol in drowning deaths may reflect the differences in alcohol policy and consumption in these countries.

In our study, 54% of boating fatalities were alcohol positive, a percentage that is comparable with a study from the USA [42]. The relative risk of dying in boating incidents increases with higher blood alcohol level and is increased 16-fold at a BAC of 1.0 g/l [42]. Legislation may affect the use of alcohol when driving a boat. In Sweden, until 2010 the legal limit for alcohol in drivers of larger boats (greater than ten metres) was 0.5 g/l; later this limit was lowered to 0.2 g/l, which is the same as for drivers of motor vehicles in road traffic. At present, there is no limit for alcohol in drivers of smaller recreational boats (less than ten metres) although it seems obvious this should be implemented.

For road traffic, it is well known that alcohol impairs a person’s judgment, performance, and behaviour [43,44] and increasing levels of impairment are associated with increased BAC levels [44]. Moreover, alcohol affects coordination and increases the risk of hypothermia [45]. Therefore, reducing alcohol use in combination with water activities may prevent many of these deaths [46].

In the present study, alcohol was found in 24% of suicidal drowning and alcohol may also have facilitated suicide. It has been reported that alcohol lowers the threshold for suicide; the presence of alcohol was 23% for suicidal drowning death compared to 38-64% for more violent suicide methods [47]. Consequently, heavy drinkers with a history of mental illness might be at higher risk of suicide [35,48].

### Pharmaceuticals and illicit drugs

Almost 25% of those who drowned had one or more pharmaceuticals in their blood. Benzodiazepines and antidepressants were most common, especially in the older age groups. Psychoactive drugs affect cognitive function, concentration, vision, coordination, and balance depending on brain concentration, individual susceptibility, and interaction with other drugs [49]. These drugs may affect risk-taking behaviour as well as affect survival possibilities after falling in the water, i.e., swimming ability. For boating, psychoactive drugs may play a similar role as for drivers in road traffic where benzodiazepine and antidepressants have been correlated with increased crash risk [50,51]. Those who committed suicide had the highest percentage of psychoactive drugs, which probably is related to a higher psychiatric morbidity. The combination of alcohol and psychoactive drugs might have an additional effect on cognitive functions [52]. However, it is difficult to know how individuals are affected as this depends on interaction with other drugs and whether higher dosages than therapeutic recommendations were used. In this study, illicit drugs were detected in almost 10% of the cases and it was higher in the undetermined group. However, most of the drowning deaths were not tested for illicit drugs. More extensive testing is needed to obtain more reliable data to assess the role of illicit drugs in drowning deaths.

### General prevention

The findings in this study suggest that it is important to reach middle/older age groups when designing future prevention programs, a recommendation also suggested by American and Australian researchers [13,53]. Education, knowledge, and training are important factors. The information about the danger of combining alcohol and drugs with water activities should be given to groups at high risk, i.e., males and the middle/older age groups. Eventually, it has previously been found that it is more difficult to protect adults than children from drowning [7]. Prevention strategies for adults should include a focus on swimming ability, ice prods, and personal floatation devices that could affect survival (i.e., the time in water). Immediate rescue and cardiopulmonary resuscitation by bystanders are also important factors that affect survival [54]. It is also important to emphasise passive measures such as designing bridges in such a way that persons cannot reach the edge, making it more difficult to jump or fall from a bridge.

### Strengths and limitations of the present study

To our knowledge, this is the first comprehensive study of drowning in Sweden. As the autopsy rate is high in Sweden for drowning deaths, probably only a few cases were missed. All autopsied cases of drowning in Sweden were analysed, including unintentional, intentional, and undetermined drowning. This approach provides a complete data set. As this type of thoroughness is often not the case in other studies, it is difficult to compare the figures with previous studies. In our study, we have information about alcohol, pharmaceutical drugs, and illicit drugs in femoral blood, whereas many previous studies on drowning death do not provide data on drugs [55].

Of all the drowning deaths, 18% of the cases were undetermined. According to a previous study that analysed the coding practices of injury deaths in Nordic countries, Sweden had a higher rate of undetermined cases and many of these were water related [24]. Therefore, the coding practice might underestimate drowning suicides in Sweden.

The data on alcohol in drowning deaths should be used with caution. Post-mortem microbial activity can produce misleading alcohol levels [18]. To avoid overestimation of alcohol, we have excluded decomposed bodies from alcohol analyses and the cut-off level for alcohol was set to 0.2 g/l in all cases. Therefore, our data represent a conservative estimate. Another way to limit this problem is to perform blood sampling within 24 hours after immersion [56], but this was not possible in all cases. It is difficult to compare studies since information about the proportion of decomposed bodies and source when obtaining the blood sample for alcohol measurement and toxicology screening often are missing. In the present study, most of the drowning deaths were not tested for illicit drugs, so there might be a selection bias.

Because the present study was based on register data, information regarding some circumstances could not be obtained, i.e., pre-drowning activities, swimming skills, use of personal floatation devices, and rescue attempts.

## Conclusions

In Sweden, the incidence of drowning deaths significantly decreased during the study period. The risk of drowning deaths is more frequent in males and middle/older age groups and increases with each year of age. Furthermore, drowning under the influence of alcohol is more frequent among males than females. Suicidal drowning constitutes one-third of all drowning deaths and more than half of the drowning deaths among females. Overall, alcohol, pharmaceutical drugs, and illicit drugs were common and might have initiated the incidents or influenced the outcome of the events. Therefore, preventive measures against both unintentional and intentional drowning should also target middle and older age groups, both sexes, and counteract the use of alcohol and psychoactive drugs during water activities.

## Abbreviations

BAC: Blood alcohol concentration; CI: Confidence Intervals; IRR: Incidence rate ratios.

## Competing interests

The authors declare that they have no competing interests.

## Authors’ contributions

KA designed the study, performed the data collection and analyses, and wrote the main draft of the manuscript. UB discussed the analyses and provided scientific advice and supervision for the whole study. BIS provided scientific advice and supervised the study as a whole. All the authors read and approved the manuscript.

## Pre-publication history

The pre-publication history for this paper can be accessed here:

http://www.biomedcentral.com/1471-2458/13/216/prepub
